# Antimicrobial peptides act on the rumen microbiome and metabolome affecting the performance of castrated bulls

**DOI:** 10.1186/s40104-023-00832-5

**Published:** 2023-03-09

**Authors:** Jinping Shi, Yu Lei, Jianping Wu, Zemin Li, Xiao Zhang, Li Jia, Ying Wang, Yue Ma, Ke Zhang, Qiang Cheng, Zhao Zhang, Yannan Ma, Zhaomin Lei

**Affiliations:** 1 College of Animal Science and Technology, Gansu Agricultural University, Lanzhou, 730070 China; 2 Key Laboratory of Animal Genetics, Breeding and Reproduction of Shanxi Province, College of Animal Science and Technology, Northwest A&F University, Yangling, 712100 China; 3 Institute of Rural Development, Northwest Normal University, Lanzhou, 730070 China; 4 Jingchuan Xu Kang Food Co., Ltd., Pingliang, 744300 China; 5 Gansu Huarui Agriculture Co., Ltd., Zhangye, 734500 China

**Keywords:** Antimicrobial peptides, Castrated bull, Growth performance, Metabolites, Microorganisms, Rumen

## Abstract

**Background:**

Many countries have already banned the use of antibiotics in animal husbandry, making it extremely difficult to maintain animal health in livestock breeding. In the livestock industry, there is an urgent need to develop alternatives to antibiotics which will not lead to drug resistance on prolonged use. In this study, eighteen castrated bulls were randomly divided into two groups. The control group (CK) was fed the basal diet, while the antimicrobial peptide group (AP) was fed the basal diet supplemented with 8 g of antimicrobial peptides in the basal diet for the experimental period of 270 d. They were then slaughtered to measure production performance, and the ruminal contents were isolated for metagenomic and metabolome sequencing analysis.

**Result:**

The results showed that antimicrobial peptides could improve the daily weight, carcass weight, and net meat weight of the experimental animals. Additionally, the rumen papillae diameter and the micropapillary density in the AP were significantly greater than those in the CK. Furthermore, the determination of digestive enzymes and fermentation parameters showed that the contents of protease, xylanase, and β-glucoside in the AP were greater than those in the CK. However, lipase content in the CK was greater than that in the AP. Moreover, the content of acetate, propionate, butyrate, and valerate was found to be greater in AP than those in CK. The metagenomic analysis annotated 1993 differential microorganisms at the species level. The KEGG enrichment of these microorganisms revealed that the enrichment of drug resistance-related pathways was dramatically decreased in the AP, whereas the enrichment of immune-related pathways was significantly increased. There was also a significant reduction in the types of viruses in the AP. 187 probiotics with significant differences were found, 135 of which were higher in AP than in CK. It was also found that the antimicrobial mechanism of the antimicrobial peptides was quite specific. Seven low-abundance microorganisms (*Acinetobacter_sp._Ac_1271*, *Aequorivita soesokkakensis*, *Bacillus lacisalsi*, *Haloferax larsenii*, *Lysinibacillus_sp._3DF0063, Parabacteroides_sp._2_1_7*, *Streptomyces_sp._So13.3*) were found to regulate growth performance of the bull negatively. Metabolome analysis identified 45 differentially differential metabolites that significantly different between the CK and the AP groups. Seven upregulated metabolites (4-pyridoxic acid, Ala-Phe, 3-ureidopropionate, hippuric acid, terephthalic acid, *L*-alanine, uridine 5-monophosphate) improve the growth performance of the experimental animals. To detect the interactions between the rumen microbiome and metabolism, we associated the rumen microbiome with the metabolome and found that negative regulation between the above 7 microorganisms and 7 metabolites.

**Conclusions:**

This study shows that antimicrobial peptides can improve the growth performance of animals while resisting viruses and harmful bacteria and are expected to become healthy alternatives to antibiotics. We demonstrated a new antimicrobial peptides pharmacological model. We demonstrated low-abundance microorganisms may play a role by regulating the content of metabolites.

**Supplementary Information:**

The online version contains supplementary material available at 10.1186/s40104-023-00832-5.

## Introduction

In 1950, the Food and Drug Administration (FDA) approved using antibiotics as feed additives. In 1994, the Ministry of Agriculture of China issued the “List of Allowable Species of Feed Drug Additives”, listing antibiotics as feed additives. Since then, antibiotics have been extensively used as growth promoters, substantially reducing production costs, morbidity, and mortality and promote animal production performance [1]. However, with the extensive use of antibiotics in the production of livestock and poultry, problems of antibiotic residues in animals and bacterial resistance have become increasingly severe. These issues posed immense threats to the development of animal husbandry and food safety for human consumption [2]. It is for these reasons that the European Union (2006), Japan (2008), the United States (2012), China (2019), and other countries have successively issued bans on the addition of antibiotics in feeds to prevent the damages caused by antibiotics abuse and to maintain food safety from animal sources and public health safety. Since most antibiotics are used in livestock farming, these bans have had a significant economic impact [3]. The pursuit of ecologically friendly and safe antibiotic alternatives has become a key subject of study in the farming industry [4]. In recent years, many different alternatives to antibiotics such as essential oils [5], organic acids [6], antimicrobial peptides [7, 8], probiotics [9], and bacteriocins [10] have also been studied. Many experiments have been done on pigs [11, 12], poultry [13], and other animals to test and develop these alternatives.

Antibacterial peptides are a class of small proteins with diverse structures and broad anti-inflammatory and growth-promoting functions. An advantage of using these proteins is that resistance cannot be easily developed against them and is commonly used in animal production. It was originally believed that antimicrobial peptides kill bacteria directly or indirectly, mainly through physical adsorption, rapid penetration, and destruction of the cell membrane [14]. Studies in recent years have demonstrated that antimicrobial peptides inhibit microorganisms through vital life processes like metabolism, biosynthesis, and translation [15]. Recent studies have also reported that antimicrobial peptides can trap bacteria by forming nanonets entrapping (Antimicrobial aggregates) [16]. Although there has been significant research on the mechanism of action of antimicrobial peptides, investigations on the use of antimicrobial peptides as animal additives have been limited to in vitro tests or examination of simple microbial diversity, such as using β-hairpin antimicrobial peptide in improving the average daily gain (ADG) of piglets and average daily feed intake (ADFI) and can effectively reduce diarrhea [17]. In mouse studies, antimicrobial peptides were found to protect and reduce the lethality of *E. coli* in mice by damaging the *E. coli* membrane [18]. A study reported that feeding chicks with the antimicrobial peptide microcin J25 significantly increased the body weight and was accompanied by a reduction in the *Salmonella* infection rate in feces and an increase in the length of intestinal villi and the depth of crypts [19].

In ruminants, the rumen acts as a bioreactor, enabling them to obtain nutrients from plants that humans cannot digest [20]. The rumen microbiota can directly or indirectly influence the growth and health of its host. Therefore, it is essential to determine the metabolic function of the rumen microbiome. The metabolic functions of the rumen microbiome reported to date have been primarily based on metagenomics [21] and transcriptomics [20, 22]. Few reports have integrated metagenomics and metabolomics to study the metabolic functions of the microbiome.

This study explores the answers to the following two fundamental questions through metagenomics and metabolomics studies: Can antimicrobial peptides disrupt the rumen microbiota through their antibacterial activities? Furthermore, is there a regulatory relationship between rumen microbes and metabolites, and do they contribute to growth performance and meat production? Based on these two main questions, the effects of antimicrobial peptides on rumen microorganisms and metabolites of castrated bulls will be compared. Further, the effects of the above two omics levels on the growth performances of the bull will be evaluated. Taken together, the goal of this study was to understand how the microbiome and metabolic changes brought about by antibacterial peptide feed supplementation influence growth performance and production in bulls.

## Materials and methods

### Animals, feeding management and experimental design

All experiments in this study were approved by the Animal Care Committee of Gansu Agricultural University (Lanzhou, People’s Republic of China) with approval number GSAU-Eth-AST-2022–035, and the experiments were performed according to the regulations and guide-lines established by this committee. The experiments were conducted in Huarui Ranch, Minle County, Zhangye, Gansu Province. Forty healthy Holstein bulls with no significant difference in body weight were castrated at 2 months of old. Bulls were fed a total mixed ration (TMR) consisting of corn silage and grain mixtures to meet or exceed their nutritional requirements outlined by the National Research Council (NRC 2000) [23]. At 10 months old, 18 animals (351.62 ± 4.69 kg BW) were selected and randomly distributed into two treatments, with nine replicates per treatment (3 bulls in each enclosure, and each bull were separated by a fence). The control group (CK) was fed the basal diet while the antimicrobial peptide group (AP) was fed the basal diet supplemented with 8 g/(d·head) antimicrobial peptides (50% each of cecropin and apidaecin). The Apidaecin (chemical structure: NH_2_-KWKLFKKIEKVGQRVRDAVISAGPAVATVAQATALAK) was from the patent product of Gansu Aolinbeier Biotechnology Group Co., Ltd., Patent No. CN201310067480.99 (Zhangye, Gansu, China), and the cecropin (chemical structure: NH_2_-PRVRRVYIPQPRPPHPRL) was from the patent product of Zhangye Aopu Biotechnology Co., Ltd., Patent No. CN20141065433.x (Zhangye, Gansu, China). The appropriate amount of antimicrobial peptide was accurately weighed, mixed with 1 kg corn daily, and top-dressed to the feed bunk. The pre-trial period was 30 d and the positive trial period was 270 d. According to the feeding standard of bulls, the diet was adjusted every 30 d and weighed the bulls (fasting). The basic diet for fattening cattle consisted of a TMR consisting of corn, silage, and grain (Table S1). All bulls were fed twice daily at 07:00 and 15:00. The remaining feed in the feed tank is collected at 6:00 every morning and weighed to measure the daily feed intake of each bull. During the experiment, all animals had ad libitum access to feed and free water, ensuring that they all received the same nutrient levels and management conditions.

### Sample collection

On the 270^th^ d, all experimental animals fasted for 12 h and were weighed. Next then the 18 animals were transported, without mixing treatment groups, to a commercial abattoir and slaughtered within 1 h of their arrival by bolt stunning followed by exsanguination from the jugular vein. Following slaughter, the carcass weight and net meat percentage were measured and calculated according to previously reported methods [24, 25]. A rumen tissue specimen of approximately 2 cm × 2 cm was immediately removed from the left dorsal sac, fixed in glutaraldehyde solution, and stored at 4 °C for examination under scanning electron microscopy (SEM). Then, 5 mL of the liquid and solid mixture was collected from the left dorsal sac of each test animal, transferred to sterile tubes, and immediately frozen with liquid nitrogen. Six randomly selected samples from each group were sent back to the laboratory the same day and stored at −80 °C for metagenomic and metabolomic sequencing. A further 15 mL of rumen content (18 bulls) was collected and stored in a sterilized container at −20 °C for measurement of digestive enzymes and fermentation parameters.

### Scanning electron microscope of rumen papilla

The diameter of the rumen papilla and the density of the micropapillary (microscopic bumps on the rumen papilla) were investigated by SEM. The sample preparation steps were as follows: 1 cm × 1 cm rumen tissue was washed twice with water for 5 min each. Next, the tissues were dehydrated with 30%, 50%, 70%, 80%, 90%, 95%, and 100% gradient alcohol, respectively for10 min each. The samples were lightly adhered to using a conductive adhesive, ion-sputtered, and finally imaged with a scanning electron microscope (Inspect, Hillsboro, TX, USA). Rumen papilla diameter and micropapillary density statistics (*n* ≥ 50) were performed using Image-Pro Plus 6.0 (Media Cybernetics, Bethesda, MD, USA) software.

### Determining the activity of digestive enzymes

The rumen contents and homogenate mixture were placed in an ultrasonic beater to obtain a 10% homogenization buffer. The supernatant was collected, and subsequent processed was done in accordance with the kit's (Biosino Biotechnology Co., Ltd., Beijing, China) instructions. The enzyme activities (Lipase, Cellulase, Protease, Xylanase and β-glucosidase) were determined by colorimetry assays [5].

### Determining fermentation parameters

Following animal slaughter, concentrations of volatile fatty acids (VFAs) were measured using the method described in the literature [5]. Briefly, the rumen contents were first centrifuged at 5400 r/min for 10 min. One milliliter of the supernatant was mixed with 0.2 mL of a 25% metaphosphate solution containing 2-ethylbutyrate as an internal standard and mixed uniformly in a new centrifuge tube. After cooling in an ice bath for 30 min, the reaction tube was centrifuged at 10,000 r/min for 10 min. The supernatant was passed through a 0.22-micron organic filter and stored in a 2-mL bottle for subsequent analysis. The volatile fatty acid content was determined by gas chromatography (Agilent, Palo Alto, CA, USA). The column temperature was maintained at 60 °C for 1 min, raised to 115 °C at 5 °C/min without reservation, and increased to 180 °C at 15 °C/min. Notably, the detector and injector temperatures were 260 and 250 °C, respectively.

### Metagenome sequencing and bioinformatics analysis

DNA was extracted from the rumen contents using Soil DNA Kit (MOBIO, Carlsbad, CA, USA). The concentration and purity of the extracted DNA were determined with TBS-380 fluorometer (Turner Biosystems, Sunnyvale, CA, USA) and NanoDrop 2000 (NanoDrop Technologies, Wilmington, DE, USA), respectively. The DNA was fragmented to fragments of approximately 400 bp using Covaris M220 (Gene Company Limited, Hongkong, China) for library construction. Sequencing was performed using the Illumina NovaSeq6000 (Illumina Inc, San Diego, CA, USA) sequencing platform. Sequencing adapters were removed with FAST software (version 0.20.0), and low-quality reads (length < 50 bp in length or quality value < 20 or with N bases) were removed [26]. Reads were aligned to the Bos Taurus reference genome assembly using BWA 0.7.9a (http://bio-bwa.sourceforge.net). Metagenomic sequencing data were assembled with MULTIPLE MEGAHIT (Version 1.1.2) [27]. Overlapping sequences with lengths ≥ 300 bp were selected as the final assembly results and used for further gene annotation. Metagenes were used to predict the best candidate open reading frames (ORFs) [28]. The predicted ORFs with length ≥ 100 bp were retrieved. A cluster analysis of a non-redundant gene catalog with sequence homology and 90% coverage was constructed using CD-HIT (version 4.6.1) [29]. Subsequently, representative sequences of the non-redundant gene catalog were aligned with the NCBI NR database using BLASTP (version 2.2.28 +) (the e-value cutoff for the best match was 1e −5) to obtain annotation results and species abundance degree [30]. Finally, a BLAST search (version 2.2.28 +) with an optimization criterion cutoff of 1e −5, annotated against the KEGG database [31], was performed using USEARCH (http://www.drive5.com/usearch/) CAZY annotation [32].

### Metabolome sequencing and bioinformatics analysis

Fifty milligrams of rumen contents were thawed on ice, and 500 µL of 70% methanol internal standard extract was added at 4 °C. After shaking for 3 min, the mixture was left to stand at −20 °C for 30 min, followed by centrifugation at 12,000 r/min for 10 min at 4 °C. Next, 250 µL of the supernatant is centrifuged at 12,000 r/min for 5 min at 4 °C. Next, 150 µL of this supernatant is taken in the liner of the corresponding injection bottle for data acquisition and further analysis. The metabolome of rumen contents was analyzed by ultra-performance liquid chromatography (UPLC) and Tandem mass spectrometry (MS/MS) (QTRAP® 6500+, SCIEX, Framingham, MA, USA) [33–35]. After obtaining the LC/MS data of different samples, the extracted ion chromatographic peaks of all metabolites were integrated using MultiQuant software (Applied Biosystems, Foster, MA, USA) and the MetWare database (MWDB) database, respectively. The chromatographic peaks of the metabolites in different samples were corrected by integration [36–38]. The relative concentrations of rumen metabolites were screened by FC (FC ≥ 2 and FC ≤ 0.5) and VIP (VIP ≥ 1) to identify the different metabolites. The identified metabolites were annotated using the Kyoto Encyclopedia of Genes and Genomes (KEGG) compound database, and the annotated metabolites were then mapped to the KEGG Pathway database [39].

### Data statistics and analysis

SPSS version 22.0 (IBM Corp., Armonk, New York, USA) was used for independent variance *t*-test and correlation analysis. Data are presented as mean ± standard deviation. Using ImageJ (National Institutes of Health, Bethesda, MD, USA) to calculate the papillae's diameter and the micropapillary density. Metabolome statistics were analyzed based on retention time and ion current strength using the MultiQuant software to calculate the relative content of each compound. Orthogonal projections to latent structures-discriminate analysis (OPLS-DA) were used to determine metabolic differences between the two groups. Enrichment analysis of metabolic pathways (MPEA) was performed by MetOrigin [40]. The OmicShare Tools [41] were employed to perform the two-way orthogonal partial least squares (O2PLS) analysis. Moreover OriginPro 9.1 (OriginLab, Northampton, US, USA) were used to draw statistical maps.

## Results

### Effects of antimicrobial peptides on growth performance of castrated bulls

Average daily gain (ADG), dry matter intake (DMI), net-meat weight, feed/gain ratio (F/G), and carcass weight were measured for statistical analysis (Table 1). The results showed that the ADG gain in the AP was significantly greater than that in the CK (*P* = 0.021). Carcass weight and net-meat weight was extremely significantly greater than those in the CK (*P* = 0.001). However, the DMI and F/G in the AP were greater than that in the CK, but not significantly different (*P* > 0.05).

**Table 1:** Comparative analysis of production performance

Item	CK	AP	*P*-value
ADG, kg	0.96 ± 0.07	1.17 ± 0.05	0.021
DMI, kg/d	10.45 ± 0.25	11.00 ± 0.25	0.134
Carcass weight, kg	323.26 ± 11.34	382.80 ± 11.30	0.001
F/G	9.12 ± 1.01	10.55 ± 1.09	0.349
Net meat weight, kg	258.55 ± 7.93	307.65 ± 8.07	0.001

*ADG* Average daily gain, *DMI* Dry matter intake, *F/G* Feed/gain ratio

### The effect of antimicrobial peptides on the activity of digestive enzymes

The contents of digestive enzymes were examined to compare their digestibility between the two groups (Table 2). The results showed that the lipase content in the CK was greater than that in the AP (*P* = 0.025), but the content of protease, xylanase, and β-glucosidase in the AP was vary significantly greater than that in the CK (*P* = 0.001). However, there was no different in cellulase (*P* = 0.974).

**Table 2:** Effects of antibacterial peptide on rumen digestive enzyme activities in bull

Item	CK	AP	*P*-value
Lipase, U/mg	8.50 ± 0.37	6.94 ± 0.10	0.020
Cellulase, U/m	89.36 ± 7.46	88.97 ± 6.77	0.974
Protease, U/mg	1.77 ± 0.09	2.84 ± 0.08	0.000
Xylanase, U/mg	3.92 ± 0.07	6.64 ± 0.30	0.000
β-glucosidase, U/mg	21.66 ± 0.62	32.43 ± 1.61	0.001

### The effect of antimicrobial peptides on rumen fermentation parameters

Herein, we assessed the presence of four major VFAs in the rumen (Table 3 and Table S2). The results showed that the acetate and valerate were greater than in the CK (*P* = 0.039*, P* = 0.018). Moreover, the level of propionate and butyrate in the AP was significantly greater than that in the CK (*P* = 0.004, *P* = 0.001).

**Table 3:** Effects of antibacterial peptide on rumen fermentation parameters in bull

Item	CK	AP	*P*-value
Acetate, mg/kg	20.01 ± 0.41	21.34 ± 0.38	0.039
Propionate, mg/kg	10.2408 ± 0.1909	11.52 ± 0.28	0.004
Butyrate, mg/kg	9.41 ± 0.26	11.09 ± 0.22	0.001
Valerate, mg/kg	0.67 ± 0.01	0.82 ± 0.04	0.018

### Effect of antimicrobial peptides on the rumen epithelium

**Fig. 1:**
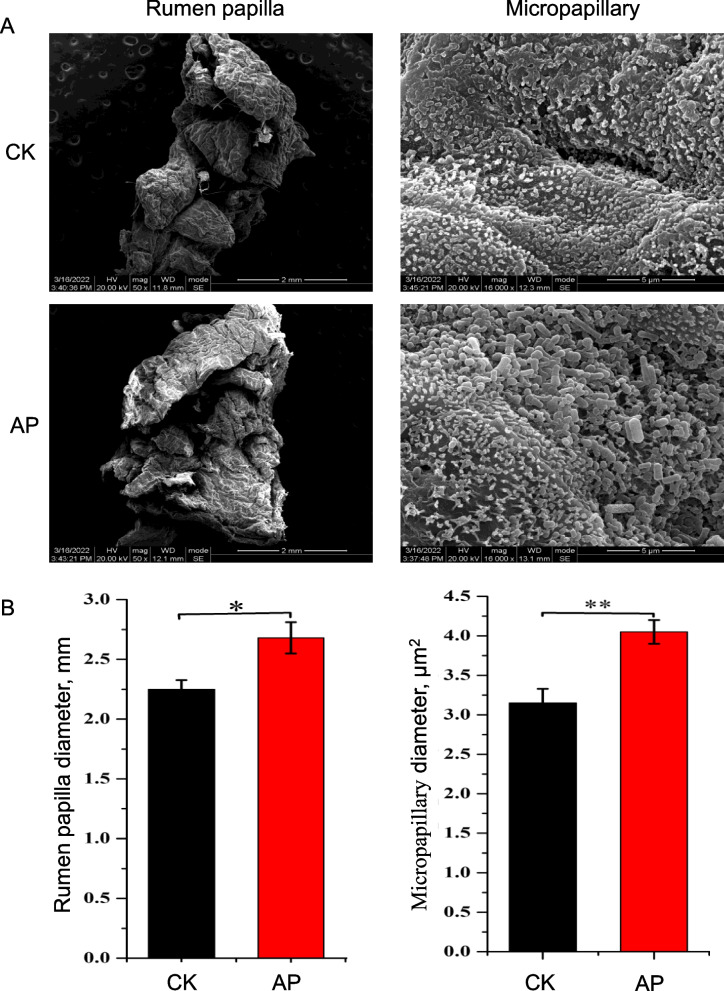
Scanning electron microscope and analysis of rumen papilla. **A** Scanning electron microscope image of rumen papilla and rumen micropapillary. **B** Rumen papilla diameter and ruminal micropapillary density. ^*^P < 0.05, ^**^P < 0.01

The growth and development of the rumen epithelium in castrated bulls were studied by scanning electron microscopy using rumen tissue (Fig. 1A and Fig. S1). From the electron microscopy images, it can be observed that the development of rumen papillae in the AP is better than that in the CK. In order to understand these changes more objectively, the ruminal papillae's diameter and the micropapillary density were analyzed (Fig. 1B). The diameter of rumen papillae in AP was larger than that in CK (*P* = 0.31). The density of the micropapillary in the AP was also more than that in CK (*P* = 0.004).

### Genome profiling of rumen microorganisms

**Fig. 2:**
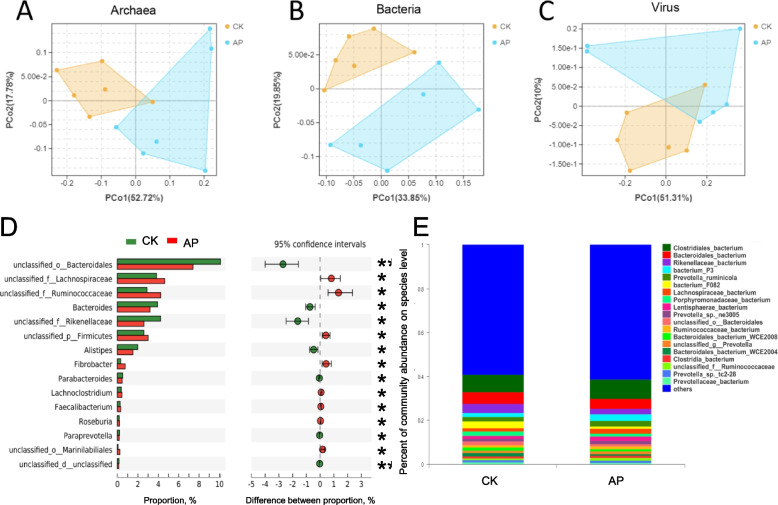
Domain-level PCoA and genus and species level microbial analysis **A** Archaea PCoA analysis chart. **B** Bacteria PCoA analysis chart. **C **Virus PCoA analysis chart. **D** Differences between CK and AP at the genus level. **E** The dominant strains of CK and AP at the species level

Metagenomic analysis of rumen contents in CK and AP showed that 74,792,855 and 70,449,158 raw reads were obtained on average. After excluding low-quality and n-containing reads, CK and AP acquired 73,283,668 and 69,227,622 clean reads, accordingly. Furthermore, optimized reads obtained for subsequent analysis after removing the host genome sequence were 61,891,630 and 58,924,630, accounting for 82.79% and 83.82% of RAW reads for CK and AP, respectively. The underlined results indicated that the sequencing data are credible and can be used for subsequent bioinformatics investigation. Archaeal (Fig. 2A), bacterial (Fig. 2B), and viral (Fig. 2C) species had isolates across the two groups, according to Principal Coordinates Analysis (PCoA) at the domain level, but no isolates with eukaryotic species or unclassified microorganisms were found. Therefore, the comparative analysis of rumen microbes between the two groups only focused on bacteria, archaea, and viruses.

### Differences in rumen microbial taxonomy

The dominant bacterial phyla in the rumen were Bacteroidetes (CK: 44.47%, AP: 38.98%), Firmicutes (CK: 36.07%, AP: 40.62%), and unclassified Bacteria (CK: 8.515%, AP: 7.011%). According to the comparative analysis of differential abundance at the genus level revealed that among the top 15 genera with significant variations in abundance, the abundance of 7 species in the CK was greater than that in the AP (*P* < 0.05), and 8 genera were significantly lower than those in the AP group (*P* < 0.05, Fig. 2D). At the species level, the dominant bacteria in the CK and AP were *Clostridiales bacterium* (CK: 8.126%, AP: 8.98%), *Bacteroidales bacterium* (CK: 5.212%, AP: 4.328%), *Rikenellaceae bacterium* (CK: 4.224%, AP: 2.596 %); bacterium P3 (CK: 1.794%, AP: 2.949%); *Prevotella ruminicola* (CK: 2.066%, AP: 2.508%) (Fig. 2E) Although these species were dominant, the abundances did not different between the two groups.

Differential analysis revealed that there were a total of 1993 distinct microorganisms. Further, the archaeal differential analysis revealed that the abundance of 32 species with differential abundance in the rumen of AP animals was greater than that of CK (*P* < 0.05), including 5 species of *Methanobrevibacter* and 2 species of *Methanosphaera* sp. The abundance of species such as 3 *Methanobrevibacter* and 2 *Methanosarcinales* in the rumen of CK animals was greater than that of AP (*P* < 0.05) (Table S3).

A comparative analysis of the bacterial differential abundance showed that there are 1691 kinds of bacteria with significant difference (*P* < 0.05). Out of these 1691 species, 826 were greater in the CK than in the AP, and 865 in the AP were greater than in the CK (Table S4).

A comparison of the differential abundance of viruses revealed that 65 viruses showed differences (*P* < 0.05). However, only 3 viruses were more abundant in the AP than in the CK, while the remaining 62 viruses were more abundant in the CK. The abundance was significantly higher than that of the AP, which indicated that antimicrobial peptides could effectively kill most viruses and that the effect was significant (Table S5).

### Functional maps and functional differences of the rumen microbiome

**Fig. 3:**
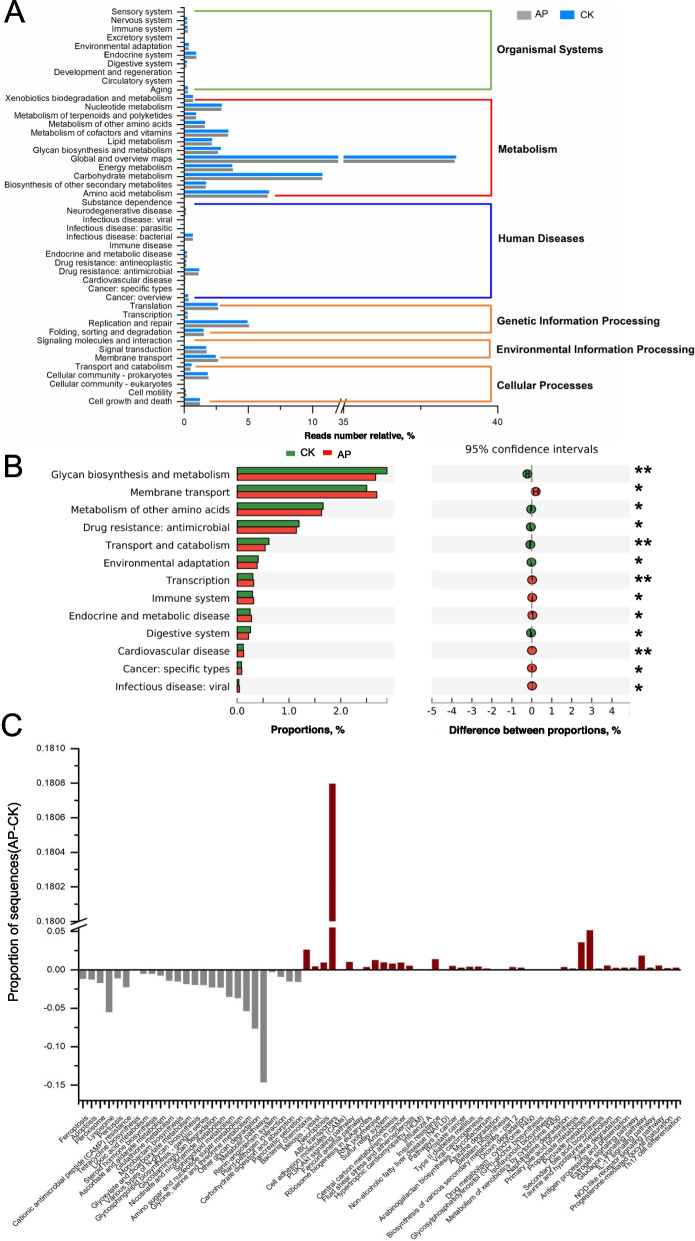
CK and AP enrichment analysis in KEGG. **A** First and second level enrichment. **B** Second level differentially significant pathway. **C** Third level differentially significant pathway

The function of the rumen microbiome was determined by KEGG map and genes encoding CAZyme. For KEGG analysis, a total of 6 pathways were annotated at the first level (Fig. 3A), namely “metabolism”, “genetic information processing”, “environmental information processing”, “cellular processes”, “organizational systems”, and “human diseases”. At the second level, 46 species were observed (Fig. 3A, Table S6). Out of these 46 global and overview maps, carbohydrate metabolism, amino acid metabolism, metabolism of cofactors and vitamins, energy metabolism, and replication and repair were the most abundant. There were 13 pathways with significant differences (*P* < 0.05, Fig. 3B). In particular, the drug resistance of the antimicrobial pathway in the CK was greater than in the AP group. In contrast, the immunological pathway was significantly higher in the AP group than in the CK, confirming that the antimicrobial peptides reduce drug resistance while enhancing immunity. 44 third-level pathways were significantly enriched in the rumen microbiota of AP bulls (Table S7, Fig. 3C): 3 “cellular process”, 4 “genetic information processing”, 3 “environmental information Processing”, 16 “Metabolic Pathways”, 11 “Human Diseases” and 7 “Organismal Systems”. On the other hand, 26 pathways were significantly enriched in the rumen of the CK. The list included 4 “cellular process pathways”, 16 “metabolic pathways”, 2 “human diseases”, and 4 “organic systems “ (Table S8, Fig. 3C).

The CAZyme map identified a total of 534 genes encoding CAZyme (Table S9), including 17 accessory activities (AA), 69 carbohydrate-binding modules (CBMs), 16 carbohydrate esterase (CE), 263 glycoside hydrolases (GH), 92 glycosyltransferases (GT) and 77 polysaccharide lyases (PL). Out of all the genes encoding CAZyme, involved in the breakdown of carbohydrates (including cellulose, hemicellulose, starch, protein, and lignin), 26 were enriched in the rumen of AP cattle (19 GH, 5 PL, 2 AA, and 1 CBM, Table S10), while 23 genes were enriched in the rumen of CK cattle (17 GH, 3 PL, 2 AA, 2 CBM, and 1 CE, Table S11). Among the GTs involved in carbohydrate synthesis, 4 were enriched in the rumen in both AP and CK.

### Comparison of probiotics and VFDB

There were substantial variations between the AP and CK for 187 probiotics. Furthermore, 135 probiotics in the AP were greater than that in the CK, especially the *Propionibacterium* and *Acetobacter* in the AP were more than 10 times higher than in the CK, on average. The 4 probiotics, *Carnobacterium*, *Ellulomonas*, *Lactobacillus*, and *Klebsiella*, were not detected in the CK but were present in the AP (Table S12). The virulence factor analysis of the Virulence Factor Database (VFDB) revealed that 364 strains produced 1151 virulence factors. The abundance of bacteria producing these virulence factors was not significantly different between the two groups, indicating that adding antimicrobial peptides did not contribute to the virulence factors while increasing the beneficial bacteria.

### Rumen metabolome analysis

**Fig. 4:**
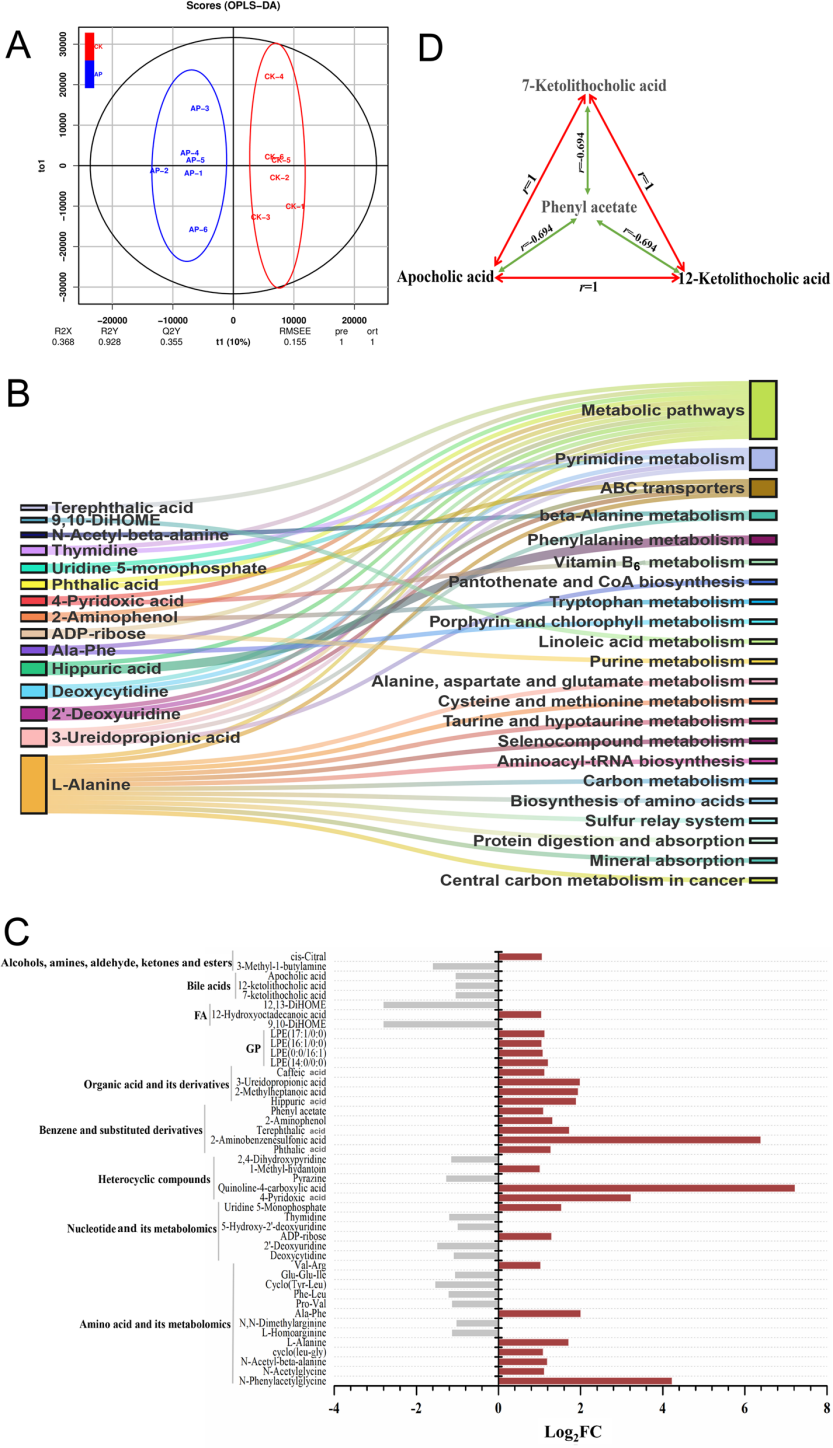
Change in rumen content metabolite levels in castrated bulls with AP diet. **A** OPLS-DA analysis. **B** Pathways of differential metabolite enrichment. **C** Differential metabolites. **D** Correlation between 7-ketolithocholic acid, apocholic acid, 12-ketolithocholic acid, and phenylacetate

A total of 662 compounds were identified in the rumen metabolites. The OPLS-DA score map showed that both groups could separate rumen metabolites (Fig. 4A). After screening the relative concentrations of rumen metabolites by FC (FC ≥ 2 and FC ≤ 0.5) and VIP (VIP ≥ 1), the expression of 45 metabolites were significantly different between the CK and AP. These included 27 upregulated and 18 downregulated metabolites (Fig. 4C). Analysis of the 45 differential metabolites showed that 14 were derived from amino acids and their derivatives, 6 were from nucleotides and their metabolites and 25 belong to others. KEGG pathway analysis revealed that of these 45 differential metabolites, 15 were significantly enriched in 22 pathways (Fig. 4B). The metabolites with significant differences were screened according to the screening criteria and analyzed by the Pearson correlation analysis method. According to the obtained results, the highest correlation coefficients of three metabolites (7-ketolithocholic acid, apocholic acid, and 12-ketolithocholic acid) were down-regulated, and one metabolite (phenyl acetate) was upregulated in the AP. It is particularly noteworthy that 7-ketolithocholic acid, apocholic acid, and 12-ketolithocholic acid showed a robust and positive correlation with each other (*r* = 1), while phenyl acetate and the above mentioned three metabolites (7-ketolithocholic acid, apocholic acid, and 12-ketolithocholic acid) showed a moderate, negative correlation (*r* = −0.694) (Fig. 4D).

### Combined metagenome and metabolome analysis

**Fig. 5:**
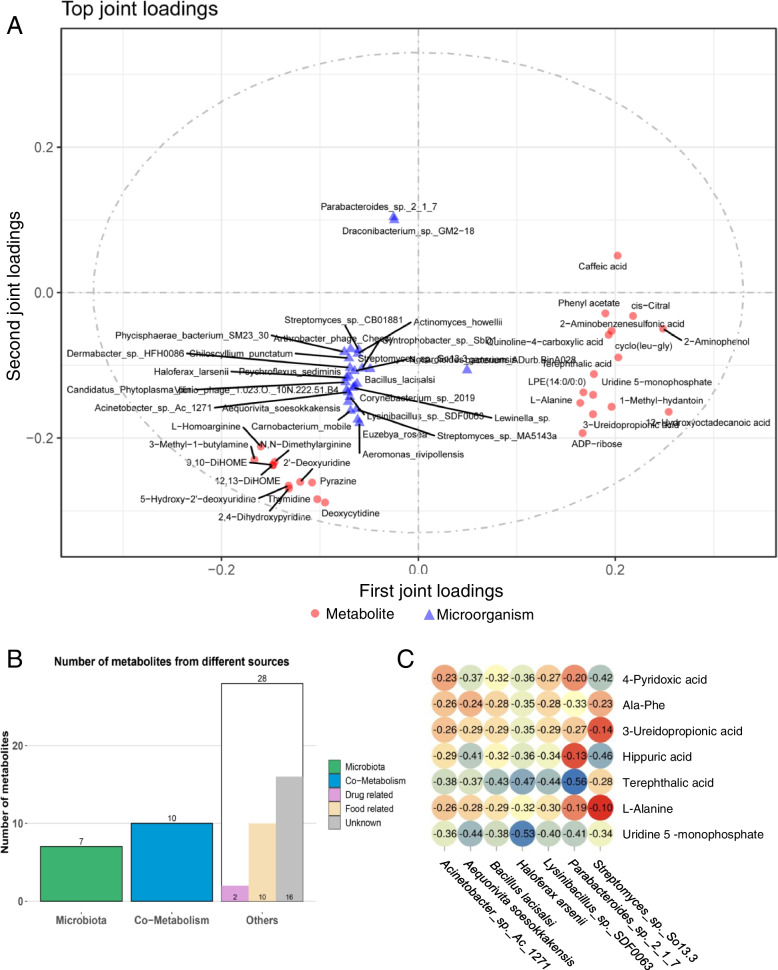
Combined metagenome and metabolome analysis. **A** Metagenome and metabolome O2PLS analysis. **B** Metabolite MPEA Analysis. **C** Correlation analysis between target metabolites and microorganisms

The data of the two omics were analyzed to examine whether there is a linkage effect between the two omics. The O2PLS analysis revealed the top 25 microorganisms and metabolites with the largest linkage effect (Fig. 5A). The data was analyzed by MPEA via MetOrigin to further screen the differential metabolites related to microorganisms. The analysis eliminated the metabolites from the host and only 17 metabolites from the microorganisms or shared by both the microorganisms and the host were retained (Fig. 5B). Top 25 microorganisms obtained by O2PLS analysis were classified, and 17 microorganisms did not have any classification. Viruses and unclassified microorganisms were removed, and only 17 microorganisms belonging to archaea and bacteria were retained. Venn diagrams of differential metabolite enrichment pathways and differential microbial enrichment pathways were explored, and it was found that four pathways are common enrichment pathways in metabolites and microorganisms (ABC transporters, Metabolic pathways, Taurine, and Hypotaurine Metabolism, Sulfur relay system). Screening 17 metabolites again with FC ≥ 2.5, 7 differential metabolites (4-pyridoxic acid, Ala-Phe, 3-ureidopropionate, hippuric acid, terephthalic acid, *L*-alanine, uridine 5-monophosphate), all upregulated, were obtained. Similarly, screening 17 microorganisms with *P* ≤ 0.005, 7 microorganisms (*Acinetobacter_sp._Ac_1271, Aequorivita soesokkakensis, Bacillus lacisalsi, Haloferax larsenii, Lysinibacillus_sp._3DF0063, Parabacteroides_sp._2_1_7, Streptomyces_sp._So13.3*) were obtained. The abundance of these seven microorganisms was down-regulated in the AP group. In order to further explore the relationship between these 7 metabolites and 7 microorganisms, a Pearson correlation analysis was done, and the results showed a negative regulatory correlation between these 7 microorganisms and 7 metabolites (Fig. 5C).

### Correlation analysis of microorganisms, metabolites, and phenotypes

**Fig. 6:**
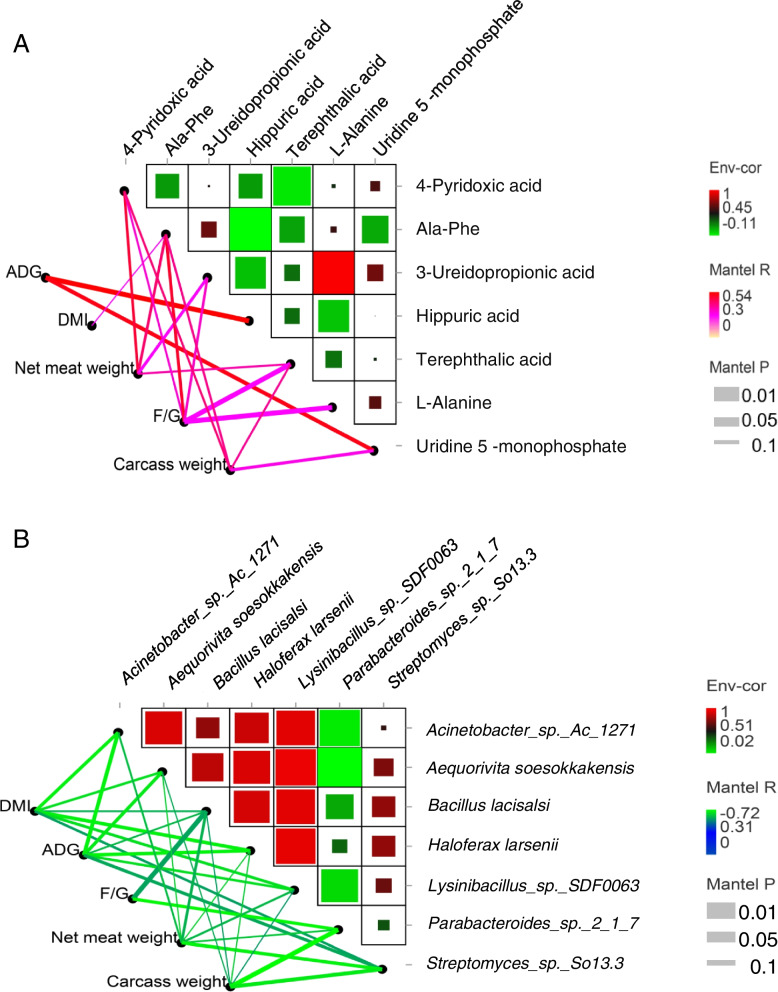
Correlation analysis between microorganisms, metabolites, and production performance. **A** Correlation analysis between metabolites and production performance. **B** Correlation analysis between microorganisms and production performance

To understand the effects of the screened seven metabolites and seven microorganisms on the growth performance of castrated bulls, a correlation analysis was performed. The results revealed that the seven upregulated metabolites showed a positive correlation (*r* ≥ 0.3, Fig. 6A) with feed conversion ratio, average daily gain, net meat weight, and slaughter rate. Especially hippuric acid and uridine 5-monophosphate showed a positive correlation with average daily gain (*r* > 0.5), Ala-Phe with feed conversion ratio (*r* > 0.5). It is also important to note that through the correlation analysis of microorganisms and production performance, a negative correlation was observed between the seven down-regulated microorganisms and dry matter intake, feed conversion rate, average daily gain, net meat weight, and slaughter rate (*r* ≤ −0.3). The *Parabacteroides_sp._2_1_7* was negatively correlated (*r* ≤ −0.7) with carcass weight, feed conversion ratio, and the average daily gain. The average daily gain was moderately negatively correlated (*r* ≤ −0.6) with *Haloferax larsenii*, *Acinetobacter_sp._Ac_1271*, and *Aequorivita soesokkakensis*. DMI and *Acinetobacter_sp._Ac_1271*, *Haloferax larsenii* were moderately negatively correlated (*r* ≤ −0.6). Furthermore, *Streptomyces_sp._So13.3*, meat weight and carcass weight were moderately negatively correlated (*r* ≤ −0.6, Fig. 6B).

## Discussion

By integrating the rumen microbiome and metabolites, the effect of antimicrobial peptides on growth performance was studied in castrated bulls. Additionally, the effect of rumen microbes and metabolites on production performance and their composition were estimated.

Acetate, propionate and butyrate, which are fermentation products of feed in the rumen, were beneficial to the growth and development of the rumen [42, 43], VFAs infusions stimulate cellular proliferation in the ruminal epithelial tissue of ruminants [44]. Interestingly, the present study established that antibacterial peptide feed supplementation increases the papillae diameter and micropapillary density of bulls, this may be correlated with high VFAs concentration in the rumen. This result is important as the rumen papilla facilitates the absorption of nutrients. Further, the content of digestive enzymes (protease, xylanase, and β-glucosidase) in the rumen contents of the AP was significantly higher than that of the CK. Xylanase can degrade xylan (a type of hemicellulose) and convert it into a monosaccharide for absorption and utilization by the body [45]. Similarly, β -glucosidase can break down cellulose into glucose to provide energy for the growth and development of the body [46]. Cellulose and hemicellulose are the primary nutrients in the rumen of ruminants broken down by xylanase and β-glucosidase into monosaccharides that can be directly absorbed and utilized by the rumen papilla. The increase in the rumen mucosal surface area (Fig. 1) promotes the absorption of these energy substances [47], contributing to the growth and development of bulls. The addition of antimicrobial peptides increased the surface area of rumen mucosa, the content of digestive enzymes and VFAs, these factors interacted and played a role together, which may be the main reason why the growth performance of AP was better than that of CK.

Similar to many previous studies that have assessed rumen microbiomes using metagenomics, bacteria were the most abundant rumen microbial kingdom in the rumen of bull and the differences in the rumen microbial features between CK and AP castrated bulls were mainly found in bacteria [38, 48]. Bacteria are key players in most of the feed biopolymer degradation and fermentation [49], which suggests that the bacteria play more significant roles in contributing to host growth performance than other microbial kingdoms. CAZyme degrades diet structural polysaccharides to provide nutrient substances for absorption by rumen epithelium. The enrichment of genes encoding CAZyme, which are involved in the breakdown of carbohydrates (GH and PL) in the rumen microbiota of AP bull, further demonstrated that the addition of antimicrobial peptides provided bulls with a greater ability to degrade complex substrates. There was no difference in the abundance of genes encoding CAZyme involved in carbohydrate synthesis (GT) in the rumen of the CK and AP. The primary role of VFAs in ruminants is to provide energy. The main function of rumen microorganisms is to decompose nutrients into VFAs and ammonia and then perform re-biosynthesis or energy metabolism. The abundance of genes encoding CAZyme involved in carbohydrate synthesis (GT) was similar in the rumens belonging to the CK and AP, but the concentration of major VFAs was higher in the AP. This result indicated that the rumen microbiota in the AP was probably more efficient in producing VFAs, thereby providing more energy for the growth and meat production of the host bull. Feed-efficient animals produce more VFAs and less methane [50]. The AP had higher VFAs content in the rumen and more methanotrophic species than the CK (CK: 12, AP: 18, Table S3), indicating that the CK may have higher feed utilization than the AP group. In fact, although the F/G of CK was higher than AP, there was no difference between the two groups (*P* = 0.349), probably because AP methanogens can use microbial fermentation to convert H_2_ and CO_2_ in the rumen into methane which is then excreted [51]. Thus, the fermentation process reduces the accumulation of H_2_ in the rumen, preventing the inhibition of enzymes involved in metabolism, promote the activity of digestive enzymes [51–53], improving AP feed utilization.

The antibacterial peptide could selectively kill microorganism extremely effectively. As a result, the AP had only three species, while the CK had as many as 62 species of virus. Furthermore, out of a total of 187 identified probiotics with different, 135 probiotics were greater in the AP than in the CK group. Especially, the abundance of *Propionibacterium* and *Cetobacter* was more than 10 times greater in the AP compared to the CK, on average. Further, four probiotics, *Carnobacterium*, *Ellulomonas*, *Lactobacillus* and *Klebsiella *were unique to the AP group (Table S12). These probiotics improve growth and development. For example, *Cetobacter* can produce vitamin B2 [54], which is associated with the metabolism of carbohydrates in the body [55]. On the other hand, *Lactobacillus* is a probiotic that is often used in livestock breeding because it promotes growth. Many studies have also confirmed that adding *Lactobacillus* can promote growth performance and ADG [56]. Based on the high efficiency of antibacterial peptides in killing viruses without affecting probiotics and even though the antimicrobial peptides reduced the abundance and species of microorganisms in the rumen, but the dominant bacteria did not change. it can be assumed that the antibacterial function of antibacterial peptides is specific. However, this assumption is contrary to the classic antimicrobial peptides pharmacodynamic model [57], which holds that antimicrobial peptides exert a microbial killing effect by broadly targeting microorganisms, thereby establishing a broad inhibitory mechanism against microorganisms. However, a recent re-evaluation of this model revealed that different antimicrobial peptides could act synergistically, exhibiting specificity toward inhibiting microorganisms [14]. In this study, equal amounts of cecropin and apidaecin were mixed to prepare the antimicrobial peptide, combined with self-produced antimicrobial peptides. This may be the main cause for the sterilization specificity of adding antimicrobial peptides. Our experimental results also confirm that the new model is correct. This study also reveals that the antimicrobial peptides improved immunity without developing drug resistance (Fig. 3B). Which is a simple and efficient killing mechanism of antimicrobial peptides, effectively preventing the evolution of bacterial resistance [14, 58], while the efficient killing of the virus directly improves immunity.

Bile acids are synthesized from cholesterol in the liver and are further hydrolyzed by bacterial bile salt hydrolase to form toxic free bile acids. These free bile acids further inhibit and kill beneficial bacteria such as *Escherichia coli* and *Bifidobacterium* bacteria [59], inhibiting growth [60]. Analysis of the metabolites revealed that 7-ketolithocholic acid, 12-ketolithocholic acid, and apocholic acid were significantly upregulated in the CK. Apocholic acid is a by-product of the bacterial metabolism of bile. It is acidic and is suspected to be carcinogenic, but the exact mechanism of its carcinogenic activity is not known. Phenylacetate is reported to inhibit these three harmful bile acids. Thus, the concentrations of the three bile acids were much lesser in the AP since the content of phenyl acetate in AP is 114% higher than CK. Many esterase can be produced by microorganisms. Esterase is the key enzyme for the synthesis of phenylethyl acetate, and acetic acid is the substrate for the synthesis of phenylethyl acetate [61]. Interestingly, phenyl acetate has antibacterial activity [62], which is essentially consistent with the results of this study.

Seven down-regulated microbes and seven upregulated metabolites that may play an essential role in the production performance of bulls were identified. It was further observed that a negative regulatory relationship existed between the metabolites and the microbes. It has been reported that feeding parabacteroides distasonis to mice can promote the production of secondary bile acids, thereby inhibiting weight gain [63]. Keystone cholic acid and Parabacteroides were significantly down-regulated in the AP, while the ADG and net meat weight in the AP were significantly higher than those in the CK. These results were consistent with those reported for mice. *Streptomyces_sp._So13.3* (*Streptomyces* genus) produces a bicyclic 19-peptide compound BI-32169, which potently inhibits the glucagon receptor and hence, can act as a glucagon antagonist [64]. Glucagon is a hormone that promotes catabolism, reduces body weight, and increases energy expenditure [65]. Furthermore, a negative correlation was found in this study between *Acinetobacter_sp._Ac_1271* and terephthalic acid because *Acinetobacter* sp. can degrade terephthalic acid [66]. It is necessary to perform further studies to determine the correlation between variety of other microorganisms and metabolites.

In order to view these bacterial populations in numerous samples, it has been standard protocol to show organisms at abundances greater than 1%, or group the low-abundant organisms into an “other” category [67–70]. While this is a widely accepted method, it may miss the key role of low abundance populations. Conjoint analysis and correlation analysis revealed that *Streptomyces_sp._So13.3* exhibited a weak correlation with ADG (Fig. 6B), *Streptomyces_sp._So13.3* also moderately correlated with hippuric acid (Fig. 5C), and highly correlated with hippuric acid and ADG (Fig. 6A). This result is consistent with reports on several other strains and metabolites. It is worth noting that the relative abundance of these strains is less than 0.0001%. Previous studies only focused on some high-abundance microorganisms that may not present the entire picture properly. This study demonstrates that metabolites may amplify low-abundance species, resulting in improved production performance. The importance of low abundance organisms has been reported in other systems. For example, in peatland communities, 0.006% of the 16S rRNA reading of desulfurization bacteria has a significant contribution to the overall sulfate reduction [71].

## Conclusion

This study identifies taxonomic features, functions, metabolites of rumen microbes, and their interactions with metabolites that contribute to host growth performance. The antibacterial mechanism of antimicrobial peptides is specific and did not affect probiotics in the rumen. Additionally, antimicrobial peptides could efficiently destroy viruses. While enhancing immunity, problems related to drug resistance are also not associated with them. Low-abundance microorganisms play a growth-promoting role by regulating metabolites. Finally, in this study, 7 microorganisms were screened that negatively regulated growth performances and 7 metabolites that had a growth-promoting effect. And we found that low-abundance microorganisms may play a role in improving the production performance of bulls by regulating metabolites.

## Supplementary Information

**Additional file 1: Fig S1.** Scanning electron microscope of rumen papilla.

**Additional file 2: Table S1.** Composition and nutrient levels of experimental diets.

**Additional file 3: Table S2.** Effects of antibacterial peptide on rumen fermentation parameters in bull.

**Additional file 4: Table S3.** CK and AP significant difference in archaea.

**Additional file 5: Table S4.** CK and AP significant difference in bacteria.

**Additional file 6: Table S5.** CK and AP significant difference in virus.

**Additional file 7: Table S6.** KEGG second level differentially significant pathway.

**Additional file 8: Table S7.** KEGG third level differentially significant pathway.

**Additional file 9: Table S8.** Pathways in which microorganisms in the CK group were significantly enriched in third level pathways.

**Additional file 10: Table S9.** CAZy map annotated genes.

**Additional file 11: Table S10.** Genes significantly enriched by CAZy in AP rumen.

**Additional file 12: Table S11.** Genes significantly enriched by CAZy in CK rumen.

**Additional file 13: Table S12.** Comparison of CK and AP probiotics.

## Data Availability

The metagenomic data are available in the NCBI database under accession PRJNA854757.

## Abbreviations

AA: Accessory activities
ADFI: Average daily feed intake
ADG: Average daily gain
AP: Antimicrobial peptide group
CAZYme: Carbohydrate-active enzyme
CBM: Carbohydrate-binding modules
CE: Carbohydrate esterase
CK: Control group
DMI: Dry matter intake
F/G: Feed/gain
FC: Fold change
FDA: Food and Drug Administration
FDR: False discovery rate
GH: Glycoside hydrolases
Gt: Glycosyl transferases
KEGG: Kyoto Encyclopedia of Genes and Genomes
NCBI: National Center for Biotechnology Information
O2PLS: Two-way orthogonal partial least squares
OPLS-DA: Orthogonal projections to latent structures discrimination analysis
PCoA: Principal coordinates analysis
PL: Polysaccharide lyases
TMR: Total mixed ration
UPLC: Ultra-performance liquid chromatography
VFA: Volatile fatty acid
VFDB: Virulence Factor Database
VIP: Variable importance in the projection
