# Supplementary figures and images for: Antimicrobial peptides act on the rumen microbiome and metabolome affecting the performance of castrated bulls
Source: J Anim Sci Biotechnol. 2023 Mar 9;14:31. doi: 10.1186/s40104-023-00832-5 (PMC9996874; doi:10.1186/s40104-023-00832-5)

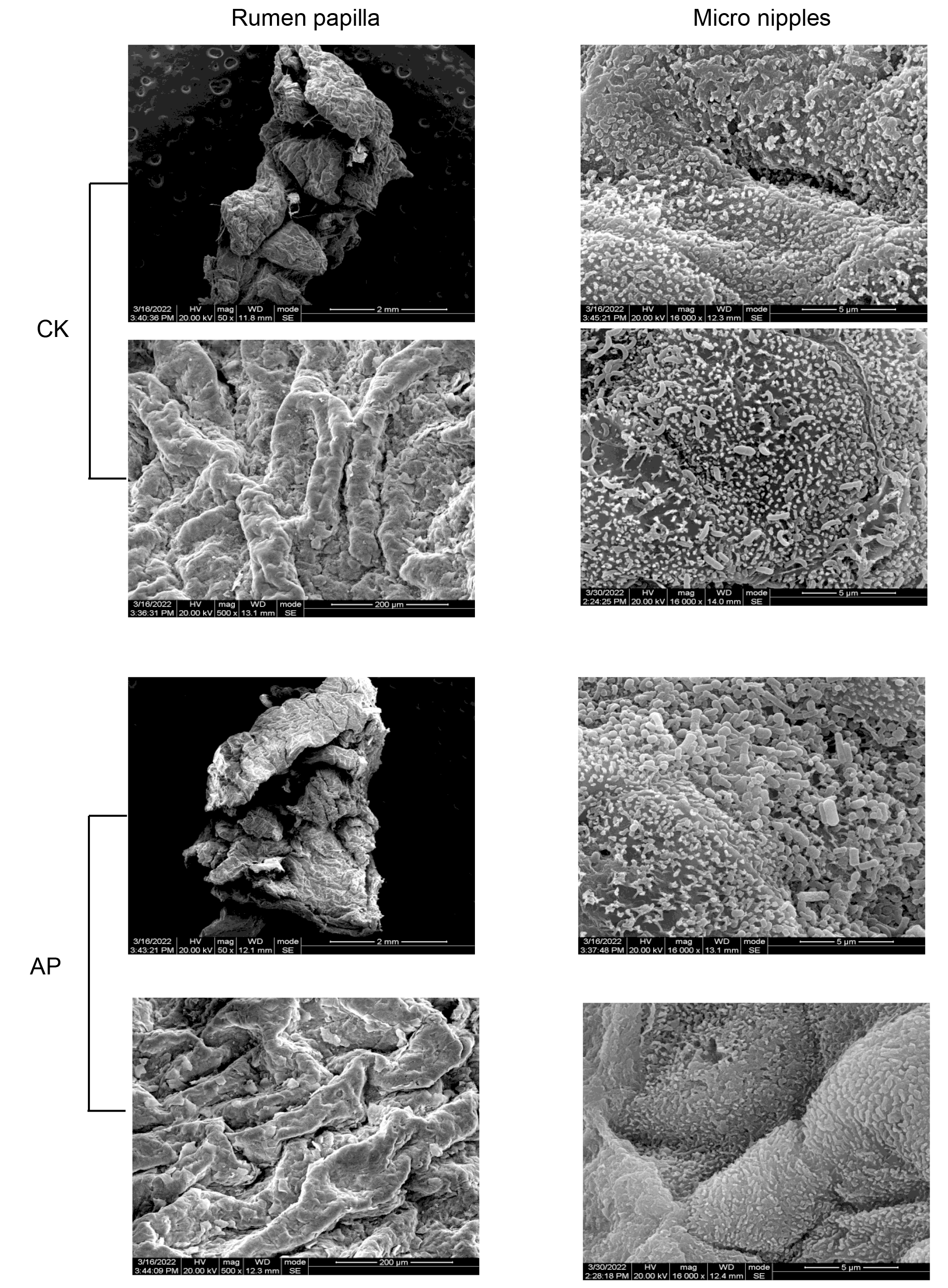

Supplement: Supplementary file 1 — Additional file 1: Fig S1. Scanning electron microscope of rumen papilla. [file 40104_2023_832_MOESM1_ESM.tif]
